# Assessing Knowledge and Practices Towards Routine Medical Checkups Among Medical Students of Karachi: A Cross‐Sectional Study

**DOI:** 10.1002/hsr2.72165

**Published:** 2026-03-23

**Authors:** Laiba Akram, Muhammad Saad Khan, Uzma Naseeb, Zarmeen Azhar, Zunnerah Akram, Muhammad Mohsin Khan, Abeeha Naqvi, Abubakr Mahmoud

**Affiliations:** ^1^ Karachi Medical And Dental College Karachi Pakistan; ^2^ Jinnah Sindh Medical University Karachi Pakistan; ^3^ Department of Biochemistry Jinnah Sindh Medical University Karachi Pakistan; ^4^ Department of Medicine University of Khartoum, ElQasr Avenue, Khartoum, Khartoum State Sudan

**Keywords:** awareness, health‐seeking behavior, non‐communicable diseases, preventive health, routine medical checkups

## Abstract

**Background and Aims:**

Non‐communicable diseases (NCDs) cause about 70% of global deaths, and early detection through routine medical checkups (RMCs) is a key preventive measure. Despite their importance, RMC uptake remains low, particularly among youth. This study assessed the knowledge, practices, and perceptions of RMCs among medical students in Karachi, Pakistan, and identified barriers to their participation.

**Methods:**

A descriptive cross‐sectional study was conducted from May to July 2025 across four medical institutions in Karachi, including JSMU, DUHS, KMDC, and SMBBMC. A total of 246 MBBS students from third to final year were selected via non‐probability convenience sampling. Ethical approval was obtained from JSMU′s Institutional Review Board (IRB No. JSMU/IRB/2025/1025). Data were collected through a structured, pilot‐tested electronic questionnaire assessing independent variables (age, gender, year of study, knowledge, attitudes, and perceived barriers) and outcome variables (practice of RMCs). Cronbach′s alpha for knowledge and practice domains was 0.76 and 0.72, respectively. Data were analyzed using SPSS v26, employing descriptive statistics, independent t‐tests, and ANOVA at a significance level of *p* < 0.05.

**Results:**

Among 246 respondents, 83% were female and the majority (77.2%) were aged 21–23 years. While 90.2% correctly identified the preventive purpose of RMCs, only 21.4% personally scheduled checkups when asymptomatic. Most students (89.9%) supported including mental health screenings in RMCs. Female gender, age group, and year of study were not significantly associated with knowledge scores (*p* ≥ 0.05). Major barriers included lack of awareness (27.2%), financial constraints (25.8%), and fear of discovering illness (16%). Although 80.4% had recommended RMCs to others, only 26.8% had undergone a checkup within the past year.

**Conclusion:**

Despite good theoretical knowledge and positive attitudes toward RMCs, actual practice among medical students remains poor. Financial barriers, perceived invulnerability, and time constraints hinder participation. Integrating preventive health practices into the curriculum, along with awareness campaigns and affordable services, may bridge the knowledge–practice gap.

## 1 Introduction

The primary cause of death and disability‐adjusted life years (DALY) lost globally is non‐communicable diseases (NCDs). According to the latest WHO estimates, NCDs kill 2000 people daily and 40 million people annually, accounting for almost 70% of all deaths worldwide [1]. Although previously regarded as “diseases of affluence,” the burden of NCDs has moved in recent years to low‐ and middle‐income countries. Cardiovascular illnesses (48%), malignancies (21%), chronic respiratory diseases (12%), and diabetes (3.5%) are the leading causes of death among NCDs. Globally, the rising frequency of NCDs necessitates public health interventions. The socioeconomic burden of addressing these chronic illnesses poses a significant issue for most countries. NCD disease burdens can only be lowered through primary prevention and early detection, which routine medical checkups (RMC) may provide [2]. RMC are visits to healthcare providers to evaluate a person′s health and risk factors for disease. They are a preventive medicine strategy that evaluates well‐being, lowers mortality and morbidity, and aids in the prediction of future illness risk factors [3, 4]. According to a 2022 study that analyzed the available data, checkups were linked to higher rates of cancer screening (e.g., colon and cervical cancer) and the identification of chronic illnesses (such as depression and high blood pressure). In addition to improving patients' self‐rated health, examinations may encourage a more nutritious diet and more frequent exercise. Checkups are not linked to lower death rates or fewer cardiovascular events like heart attacks and strokes, but they do seem to help control cardiovascular disease risk factors like high blood pressure and high cholesterol [5]. Understanding and exposing the obstacles to RMCs for the general public is therefore crucial. The survey conducted in Rawalpindi found that the most frequent excuse for not having an RMC was the higher expense of these RMCs. Laziness, a hectic schedule, the belief in being healthy, and the perception that RMCs are a waste of money are some other reasons why people do not get an RMC, according to Fazal et. al's study [2]. A study on Saudi adults found that crowded healthcare, lack of patient knowledge, financial issues, and higher‐socioeconomic status contribute to less prevalence of RMCs [6]. A study conducted in Iowa, US, discovered that financial concerns reduce the risk of having RMCs, whereas higher‐socioeconomic class persons have an increased likelihood [7]. Non‐attendance in Northern Taiwan is influenced by factors such as health beliefs, time availability, complicated screenings, bad emotions, and past experiences [8]. Routine medical check‐ups promote health and early diagnosis of chronic diseases, which improves well‐being and quality of life. While they do not reduce mortality or cardiovascular events, they do improve chronic illness detection, treatment, risk factor management, preventive service utilization, and patient outcomes [9, 10].

A study conducted in Rawalpindi indicated that 92.4% of participants acknowledged that timely diagnosis through RMCs can facilitate early treatment, and women were more generally aware of RMCs than men [2]. A survey was also done in Saudi Arabia which revealed that 69.57% of the subjects understood what RMCs were [11]. Both research participants stated that their “concern about health” was the most frequent reason for having an RMC [2, 11]. In a different Nigerian study that focused on healthcare workers' utilization of periodic exams, it was found that while 92.8 percent of participants knew a lot, just 46 percent actually performed routine checkups [12]. While 61.3% of participants in Uganda reported having heard of routine health check‐ups [3], only 24% of respondents in Northern Vietnam reported having regular health check‐ups in the previous 12 months. Their results were far lower than the rate of 51.2% found in comparable urban studies in Vietnam [13, 14].

One special group that connects professional medical knowledge with lay understanding is medical students as there is no data on preventative health behavior among medical undergraduates, especially in South Asia, despite research evaluating RMC awareness in middle‐aged populations. Evaluating their knowledge and practices offers valuable information about the preventive health practices of future medical professionals, which can have a direct impact on their patients and communities. Their exposure to preventive medicine curriculum, in contrast to the general public, enables assessment of whether such training translates into personal health practices—an area that is still understudied in Pakistan and similar lower‐middle‐income countries (LMICs). Health practitioners can benefit from this study in that it can help them understand the obstacles to regular medical examinations, identify information gaps, and create focused educational programs. This data can help address particular issues in Karachi and guide the creation of health policies. This study aims to evaluate awareness of RMC among medical students, its association with education, and factors preventing and encouraging its practice in Karachi, thereby improving the overall understanding of RMC.

## 2 Materials and Methods

### 2.1 Study Design and Setting

This was an observational descriptive study with a cross‐sectional approach conducted among medical students from multiple institutions, including the Department of Bachelors in Medicine and Bachelors in Surgery (MBBS) at Jinnah Sindh Medical University (JSMU), Karachi Medical and Dental College (KMDC), Dow University of Health Sciences (DUHS), and Shaheed Mohtarma Benazir Bhutto Medical College (SMBBMC) in Karachi, Sindh, Pakistan. The study was carried out from May 2025 to July 2025. These institutions were selected due to their prominence in medical education and their diverse student populations, offering a comprehensive representation of medical undergraduates in the region. The inclusion of students from multiple medical colleges allowed for broader insights into the knowledge, attitudes, practices, and barriers related to RMC among future healthcare professionals.

### 2.2 Participants

The study involved 3rd to Final year students from the MBBS department of JSMU, KMDC, DUHS, & SMBBMC, as these students begin their clinical ward rotations from third year onward, providing them with relevant exposure and insight into the importance and practice of RMC, having completed the basic medical sciences component of the MBBS curriculum. Students enrolled in programs other than MBBS, as well as those in the first and second years of the MBBS program, were excluded from the study due to limited or no clinical exposure and insufficient academic foundation related to preventive healthcare practices.

### 2.3 Sample Size

A non‐probability convenience sampling technique was employed to select participants from the study population. The sample size of 246 was calculated using the OpenEpi, Version 3 open‐source calculator for proportions. The calculation was based on the following parameters:

- Population size (N): 1,000,000 (for finite population correction)
- Prevalence of knowledge regarding RMC (p): 80% [2]
- Confidence level: 95%
- Margin of error (d): ±5%
- Design effect (DEFF): 1 (as this was not a cluster survey)

The formula used was:

n=[DEFF*Np(1‐p)]/[d2/Z21‐α/2*(N‐1)+p*(1‐p)]

Hence, the calculated sample size was 246.

### 2.4 Data Collection and Variables

After obtaining approval from the institute′s ethical review board, International Scientific Committee, (JSMU/IRB/2025/1025) to conduct the research and obtain informed written consent from the participants, data were recorded using an electronic, structured and standardized questionnaire by medical students (Supporting File). A pilot study with 30 participants was conducted, and the questionnaire's internal consistency was examined. Cronbach's alpha gave a value of 0.76 for knowledge and 0.72 for practice, with a cut off limit of 0.7. Knowledge, attitudes, practices, and perceived barriers toward RMC among medical students were assessed through this questionnaire. The first section gathered demographic information, including the participant's name, age, gender (male, female, prefer not to say), and current educational level (third year, fourth year, final year). The second section evaluated general knowledge related to RMC, such as how frequently should adults take RMC, the primary purpose of checkups, and the age at which routine medical checkup is generally recommended. The third section assessed understanding of preventive health measures, including the role of routine checkups in early detection of chronic diseases and the importance of mental health screening. The fourth section explored personal practices and attitudes, such as whether students regularly schedule checkups, their confidence in discussing checkups with patients during clinical rotations, and the influence of medical education on their behavior. It also inquired about factors influencing decision‐making, such as time constraints, financial concerns, or perceived need. Lastly, in the fifth section, the questionnaire investigated perceived barriers to undergoing RMC and included suggestions on how healthcare providers could promote greater uptake through interventions like health campaigns, affordable services, and flexible appointment scheduling.

### 2.5 Statistical Analysis

Data were cleaned, categorized, and analyzed using IBM SPSS Statistics for Windows, Version 26 (2017; IBM Corp., Armonk, NY, USA). A descriptive research design was employed. Independent variables associated with the outcome variable at a p‐value of < 0.05, as well as those considered of public health importance based on prior strong evidence, were selected as candidate variables. Independent *t*‐tests and one‐way ANOVA were used to assess group differences. Descriptive statistics, including means and standard deviations (SD), frequencies, percentages, minimum, and maximum values, were calculated as appropriate. The reliability of the questionnaire was evaluated using Cronbach′s alpha, with separate values reported for the knowledge and practice domains.

## 3 Results

**Figure 1:**
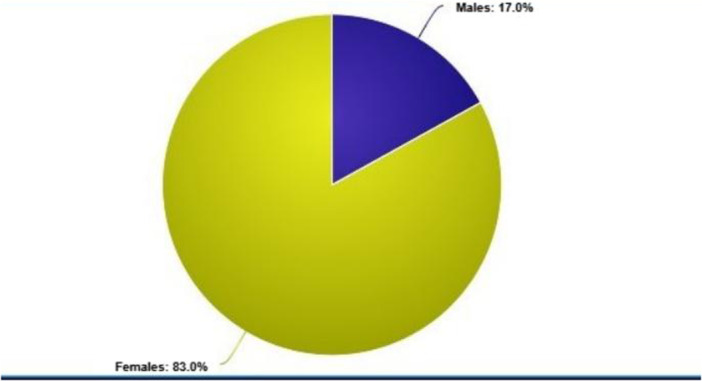
Distribution of participants by gender.

**Figure 2:**
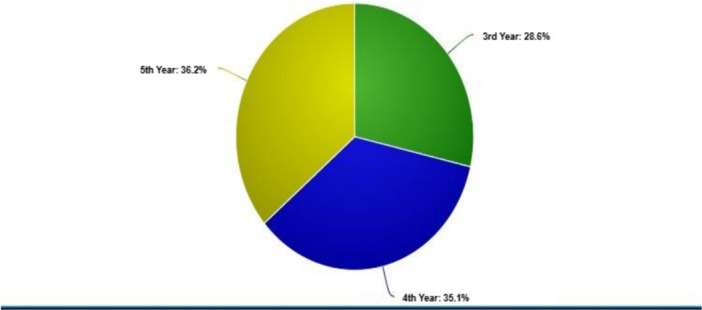
Distribution of participants by year of study.

Among 246 responses, 83.0% were female (n = 204, 95% CI: 0.79–0.87) and a considerably lesser proportion were male (*n* = 42) (Figure 1). Concerning age, the majority of the participants (*n* = 190) 77.2% were in the age bracket 21–23 years and the rest 7.6% (*n* = 19) and 15.2% (*n* = 37) in the age brackets 24–26 years and 18–20 years respectively. The moderate value of the age category was 1.08 (SD = 0.473, 95% CI: 1.02–1.13) (Table 1). The responders were more or less fairly distributed by academic year, with third‐year students accounting for 28.6% (*n* = 70), fourth‐year students for 35.1% (*n* = 86), and fifth‐year students for 36.2% (*n* = 90). The average measure for the year of investigation was 1.08 (SD = 0.803, 95% CI: 0.98–1.17) (Figure 2).

**Table 1:** Demographic Information of the Participant.

	Frequency (*n*)	Percentage (%)	Mean (SD)	95% Confidence interval CI (%)
Sex				
Female	204	83.0%	0.83 (0.377)	0.79–0.87
Male	42	17.0%		
Age				
18–20	19	7.6%	1.08 (0.473)	1.02–1.13
21–23	190	77.2%		
24–26	37	15.2%		
Year of study				
3rd Year	70	28.6%	1.08 (0.803)	0.98–1.17
4th Year	86	35.1%		
5th Year	90	36.2%		

**Figure 3:**
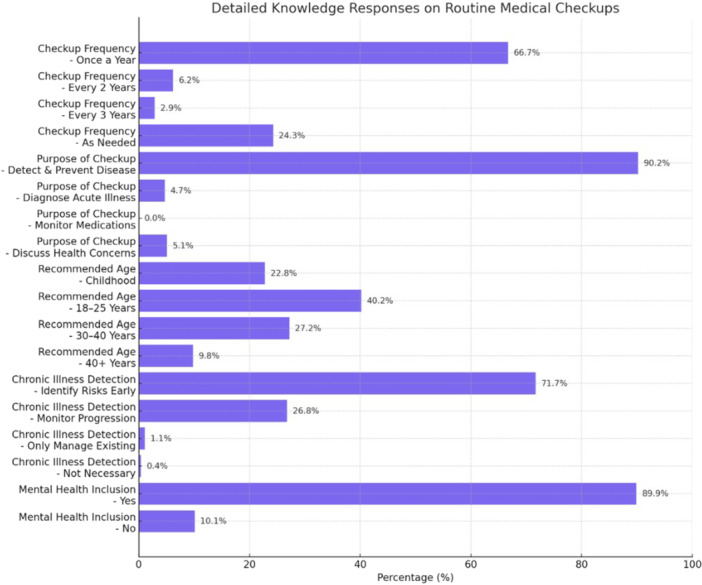
Knowledge of undergraduate medical students about routine medical checkups.

In relation to knowledge about routine medical check‐ups, the results indicate that 66.7% of the respondents identified the knowledge that adults are expected to do routine check‐ups at least once in a year. Their conceptual knowledge was high and included some understanding of the true purpose of these examinations, which is to be able to identify any type of health condition early and prevent diseases in the future 90.2%. But when it came to proper identification that regular check‐ups are to be initiated at the age of 18–5 years, only 40.2% were accurate and 71.7% knew that regular check‐ups were indeed useful in detecting any chronic illness even before its symptoms were alerted. Positively, 89.9% of the students thought that mental health screening should be included in the common routine check‐up policy indicating that they understand holistic health (Table 2 & Figure 3).

**Table 2:** Knowledge of undergraduate medical students regarding routine medical checkups (*N* = 246).

	Frequency (*n*)	Percentage %
How frequently should adults attend routine medical checkups to maintain overall health?		
Once a Year	164	66.7%
Every two years	15	6.2%
Every three years	7	2.9%
As needed	60	24.3%
What is the main purpose of attending a routine medical checkup?		
To diagnose and treat acute illness	12	4.7%
To detect health conditions early and prevent future diseases	221	90.2%
To monitor medications only	0	0
To discuss personal health concerns	13	5.1%%
At what age is it generally advised to begin regular medical checkups for adults?		
Childhood	56	22.8%
18–25 Years	99	40.2%
30–40 Years	67	27.2%
40+ Years	24	9.8%
How do regular checkups help in identifying chronic illnesses like diabetes or hypertension?		
They help monitor the progression of existing conditions	66	26.8%
They help to identify health risks before symptoms appear	176	71.7%
They only manage current conditions	3	1.1%
They are not necessary for chronic conditions	1	0.4%
Do you believe that mental health screenings should be included in standard routine checkups?		
Yes	221	89.9%
No	25	10.1%

The descriptive statistics revealed the moderate levels of knowledge among participants, as the value of mean scores in terms of general checkup knowledge and the importance of preventive care understanding comprised 1.97 (out of 3) and 1.61 (out of 2), respectively. Such scores indicate that students possess basic knowledge, but still, they can advance their level of overall comprehension (Table 3).

**Table 3:** Descriptive statistics for the measured knowledge and practices of respondents toward routine medical checkups (*N* = 246).

	Mean (SD)	Min and max score
Knowledge of general checkups	1.97 (0.790)	0‐3 POINTS
Importance of preventive care	1.61 (0.545)	0‐2 POINTS

The results of the comparison of knowledge scores by demographic characteristics showed no statistically significant difference between specialties in terms of sex, age category or year of study, showing that there is no difference in the knowledge level by the subgroups. It indicates that the demographic variables did not affect the output levels of knowledge in this sample significantly (Table 4).

**Table 4:** Comparison of mean values of the knowledge of respondents towards routine medical check‐ups in relation to their personal characteristics (*N* = 246).

	Mean (SD)	Test statistics	P‐value
Sex (t)			
Female	3.58 (0.991)	0.227	0.82
Male	3.62 (1.033)		
Age (f)			
18–20	3.57 (0.746)	0.078	0.93
21–23	3.58 (1.00)		
24–26	3.64 (1.10)		
Year of study (f)			
3rd Year	3.57 (1.046)	0.607	0.55
4th Year	3.52 (1.052)		
5th Year	3.67 (0.90)		

**Figure 4:**
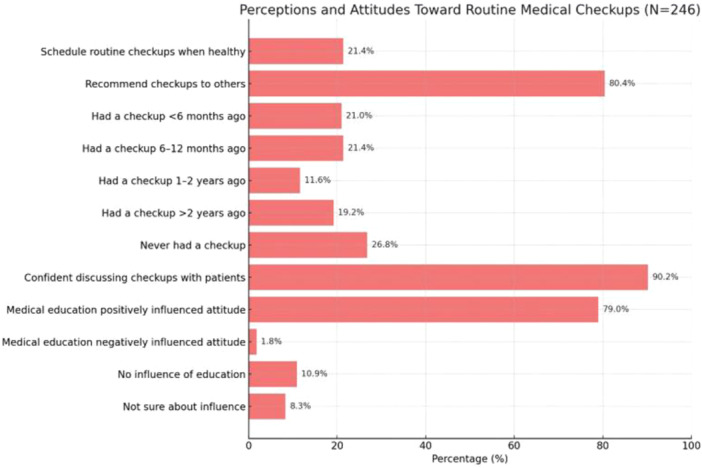
Perceptions and attitudes of undergraduate medical students toward routine medical checkups.

When examining the practices and attitudes, it was shown that only 21.4% of the students booked regular checkups when they felt well, indicating a substantial discrepancy between their knowledge and personal health behaviors. However, a report of 80.4% stated that they always advocated for checkups when speaking with friends and family, indicating a more assertive approach to giving advice. Only 26.8% of the respondents had never taken themselves through a routine medical examination. On the contrary, 90.2% of them had confidence in addressing the benefits of frequent checkups in clinical visits, whereas 79% of them stated the aspect of medical school positively affected attitudes about routine checkups. Concerning the factors hindering the seeking of routine checkups, ignorance 27.2% was cited as the most frequent hindrance to patching up in line with other barriers namely, lack of money 25.8% and fear of finding out about health issues 16%. The most important issues raised by these findings regarding the possible areas of intervention and education that can help with existing misconceptions and increase utilization rates are mentioned (Table 5, Figure 4).

**Table 5:** Practices and perceptions of undergraduate medical students toward routine medical checkups (*N* = 246).

	Frequency (*n*)	Percentage (%)
Do you personally schedule regular routine checkups even when you feel healthy?		
Yes	53	21.4%
No	193	78.6%
Have you ever recommended routine checkups to family or friends as a preventive health measure?		
Yes	198	80.4%
No	48	19.6%
When was the last time you had a routine checkup?		
Less than 6 months ago	52	21.0%
6–12 months	53	21.4%
1–2 years ago	29	11.6%
More than 2 years ago	47	19.2%
Never	65	26.8%
Do you feel confident in discussing the benefits of routine checkups with patients during your clinical rotations or practice?		
Yes	222	90.2%
No	24	9.8%
Has your medical education influenced your attitude towards routine medical check‐ups?		
Yes, positively	194	79.0%
Yes, negatively	4	1.8%
No influence	27	10.9%
Not sure	21	8.3%
What factors influence your decision to schedule or not schedule a routine checkup?		
Health concerns or symptoms	175	24.2%
Lack of time	152	21.0%
Lack of health insurance or cost concerns	98	13.6%
No perceived need (feel healthy)	141	19.5%
Forgetfulness	97	13.4%
Availability of healthcare facilities	60	8.3%
What do you think are the main barriers to routine medical checkups?		
Lack of time	116	15.8%
Lack of awareness about the importance	200	27.2%
Financial constraints or insurance issues	190	25.8%
Fear of discovering health problems	118	16.0%
Lack of healthcare providers in the area	43	5.8%
Lack of trust in the healthcare system	69	9.4%
How can healthcare providers encourage the general population to undergo regular medical checkups?		
Offering reminders or health campaigns	181	27.6%
Providing convenient and flexible appointment times	169	25.7%
Offering affordable or free checkups	115	17.5%%
Providing more information about the benefits of routine checkups	96	14.6%
Reducing waiting times at healthcare facilities	96	14.6%

Lastly, examining the connection between the level of knowledge and attitude or habits, a statistically significant distinction was monitored when it comes to confidence in speaking about checkups, in which, the students who were confident scored better with respect to knowledge levels (*p* < 0.001). No major differences were however recorded in terms of actual scheduling practices or recommendation behavior, which helps in determining that confidence in communication and its implication may be better determinant of knowledge than personal health behaviors (Table 6).

**Table 6:** Comparison of mean values of the knowledge of respondents towards routine medical check‐ups in relation to their attitude (*N* = 246).

	Mean (SD)	Test statistics	P‐value
Do you personally schedule regular routine checkups even when you feel healthy?			
Yes	3.58 (0.999)	−0.57	0.96
No	3.59 (0.967)		
Have you ever recommended routine checkups to family or friends as a preventive health measure?			
Yes	3.64 (0.947)	1.675	0.10
No	3.37 (1.062)		
When was the last time you had a routine checkup?			
Less than 6 months ago	3.73 (0.952)	1.281	0.28
6–12 months	3.61 (1.054)		
1–2 years ago	3.54 (0.962)		
More than 2 years ago	3.72 (0.886)		
Never	3.38 (0.973)		
Do you feel confident in discussing the benefits of routine checkups with patients during your clinical rotations or practice?			
Yes	3.67 (0.936)	4.177	< 0.001
No	2.84 (0.987)		
Has your medical education influenced your attitude towards routine medical check‐ups?			
Yes, positively	3.64 (0.935)	1.733	0.16
Yes, negatively	2.80 (1.483)		
No influence	3.52 (1.051)		
Not sure	3.35 (1.040)		

## 4 Discussion

RMC play a vital role in early detection and prevention of various diseases, thereby contributing significantly to public health outcomes [2]. This approach is especially significant in low‐middle‐income countries (LMIC) where the burden of noncommunicable diseases (NCD) is notably high and requires timely interventions [15]. Pakistan being a part of LMIC, having a population of 207.7 million, being the fifth most populated country is facing a double burden of both communicable and non‐communicable diseases [16, 17]. Furthermore, among medical students, understanding and adherence to such practices are crucial, as they are future healthcare providers who will counsel patients on preventive care. This study therefore aims to assess the knowledge and practices concerning RMC among medical students in Karachi, a major urban center in Pakistan.

The majority of the medical students (66.7%) have a high level of knowledge that adults are expected to do RMC at least once a year. This aligns with previous research conducted among students and staff of the Ahmadu Bello University (ABU), Zaria Medical Center, who found that a notable number of participants were aware of RMC [18]. Despite these promising statistics, the knowledge of medical students regarding RMC is still basic. The value of mean scores in terms of general checkup knowledge and the importance of preventive care understanding comprised 1.97 (out of 3) and 1.61 (out of 2), respectively. However, the students who had confidence in speaking publicly about checkups scored better with respect to knowledge levels *p* < 0.001. This suggests a need to deepen their overall understanding. This can be due to various reasons, however, according to a study conducted in Nepal, the importance of two‐way communication between students and doctors is very important to overcome the educational and hierarchical barriers that impede the students from asking important information [19]. One cultural difference is that doctors are more important than patients, and teachers are more important than students [20]. This is always seen in Asian countries which further creates a gap in two‐way communication, hence impacting the level of learning in students [20].

79% of students in our study consider that medical school has resulted in a positive impact on their attitudes about RMC. Better knowledge regarding RMC indicates better health literacy among medical students that can be due to their better access to and utilizing the information to make better healthcare‐related decisions. Hence, medical students in contrast to students of other health science disciplines have more knowledge regarding clinical medicine [19, 21].

The sociodemographic information indicated the majority of the participants being women (83%) which correlates with the finding that female students acquire 80%–85% of seats in medical colleges of Pakistan [22]. Despite this notable difference, demographic factors such as sex (*p* = 0.82), age (*p* = 0.93) and year of study (*p* = 0.55) do not affect the output level of knowledge in our study. These findings are further supported by a study conducted on healthcare workers of a tertiary hospital in Calabar, Nigeria [12]. However, this is in contrast to other studies, some of which considered male students to have a higher health literacy score than female students such as in China and Nepal while in Denmark and the USA female students scored higher. This is mainly because of cultural differences in different countries [19, 23, 24, 25].

Looking at the students' habits and beliefs, it was found in our study that only 21.4% scheduled regular checkups when they were feeling good, showing a big gap between what they knew about health and how they behaved in their own lives. According to a study, this can be due to various reasons including stress or anxiety, lack of time, fear or embarrassment of finding out any disease, poor communication between doctors and patients and lengthy waiting time [18].

The greatest obstacle encountered in seeking RMC includes ignorance (27.2%). This finding is concurrent with other surveys in which laziness is considered as one of the biggest obstacles [11]. Another important factor includes cost or lack of money. Pakistan, being a low‐middle‐income country, its healthcare is mostly paid for out of pocket, which puts a lot of financial strain on people and families [26]. According to research, cost is also one of the most important impeding factors in the Kingdom of Saudi Arabia [27]. Nevertheless, a considerable body of research has demonstrated that health insurance is connected with increased utilization of health services, particularly routine checks [28, 29].

The study has several limitations. First, the study used convenience‐based sampling, including only a small sample size of MBBS students of public sector universities majority of which are females (gender bias), hence the results cannot be generalized. However, the study does provide complete insight into a specific group which includes medical students of Karachi in this regard. Secondly, the study design limits the assessment of causal relationship between outcome and predictors. Thirdly, the desirability bias (The propensity to produce responses that are perceived to be preferable rather than those that reflect true knowledge, attitudes, or actions). Nevertheless, the study conducted a pilot study beforehand to limit any such bias. Lastly, social and familial factors that could further explain behavioral variances, such as a family history of chronic illness or previous exposure to health examinations, were not examined in this study. Further research that takes these factors into account may provide more profound behavioral insights.

Despite these limitations, this novel, multicentric study has made contributions to the utilization of health services in Karachi, Pakistan. According to a study, research knowledge is poor in postgraduate trainees of Pakistan [30]. Hence, this research provides important literature regarding the use of RMC across a wide range of factors. The strengths of the study include the measurement of both knowledge and attitudes, the use of several indicators to measure them and the examination of the statistical relationships among the variables. This provides a more complete understanding of the knowledge‐practice gap among medical undergraduates and a more reliable foundation for educational and institutional policy interventions. Recommendations include integrating more practical and behavioral aspects into the curriculum and reinforcing the importance not only of checkups but also of self‐practice for the students. Efforts to reduce stigma and fear of diagnosis, to provide services that are affordable and accessible and to improve peer‐led awareness would help to close the knowledge‐practice gap.

## 5 Conclusion

This study concludes that while most medical students understand the value of routine checkups, few actually use these services without symptoms. Barriers such as limited knowledge, cost, and systemic issues persist even among future health professionals. The findings highlight a significant knowledge‐practice gap, emphasizing the need for improved health literacy through better communication, training, and culturally sensitive education. Additionally, institutional and government support to enhance access and reduce preventive care costs is crucial, not only to benefit students' own health but also to prepare them to be effective health advocates for their future patients.

## Author Contributions

**Laiba Akram:** supervision, conceptualization, methodology, writing – reviewing and editing, validation, and formal analysis. **Muhammad Saad Khan:** writing, reviewing, and editing; formal analysis. **Uzma Naseeb:** project administration, visualization, writing, reviewing, and editing. **Zarmeen Azhar:** investigation, methodology, data curation. **Zunnerah Akram:** resources, writing, reviewing, and editing. **Muhammad Mohsin Khan:** writing, reviewing, editing, investigation, methodology, data curation, and correspondence. **Abeeha Naqvi:** formal analysis and data curation. All authors have contributed to the manuscript and approved the submitted version. **Abubakr Mahmoud:** formal analysis and data curation. All authors have contributed to the manuscript and approved the submitted version. All authors have read and approved the final version of the manuscript. The corresponding author, Abubakr Mahmoud, had full access to all data in this study and takes complete responsibility for the integrity of the data and the accuracy of the data analysis.

## Funding

The authors received no specific funding for this work.

## Ethics Statement

This study was approved by the Research Ethics Committee of the JSMU′s Internal Scientific Committee (JSMU/IRB/2025/1025) and conducted following the Declaration of Helsinki, local regulations, and institutional guidelines. Written informed consent was obtained from all participants before their inclusion in the study.

## Consent

Written informed consent was taken from the participants. All authors consent to publish this work.

## Conflicts of Interest

The authors declare no conflicts of interest.

## Data Availability Statement

Data available in article Supporting Information.

## Transparency Statement

The lead author Abubakr Mahmoud affirms that this manuscript is an honest, accurate, and transparent account of the study being reported; that no important aspects of the study have been omitted; and that any discrepancies from the study as planned (and, if relevant, registered) have been explained.

## Supporting information

HSR Supporting File of RMC.
