# Supplementary material for: Assessing Knowledge and Practices Towards Routine Medical Checkups Among Medical Students of Karachi: A Cross‐Sectional Study
Source: Health Sci Rep. 2026 Mar 23;9(3):e72165. doi: 10.1002/hsr2.72165 (PMC13097485; doi:10.1002/hsr2.72165)
Supplement: Supplementary file 1 — HSR Supporting File of RMC. [file HSR2-9-e72165-s001.docx]

**Assessing Knowledge and Practices Towards Routine Medical Checkups Among Medical Students of Karachi**

**Supplementary File**

**Section 1: Questionnaire**

**Questionnaire on Assessing Knowledge and Practices Towards Routine Medical Checkups Among Medical Students:**

**Demographic Information:**

1. **Name? _________________**
2. **Age?**
3. **Gender?**
   - a) Male
   - b) Female
   - c) Prefer not to say

**4.Educational level?**

- - a) Third Year
  - b) Fourth Year
  - c) Final Year

**Knowledge and Practices on Routine Medical Checkups – Questionnaire**

**Section 1: General Knowledge**

1. **How frequently should adults attend routine medical checkups to maintain overall health?**
   - a) Once a year
   - b) Every 2 years
   - c) Every 3 years
   - d) As needed
2. **What is the main purpose of attending a routine medical checkup?**a) To diagnose and treat acute illnesses

a) To diagnose and treat acute illnesses

- - b) To detect health conditions early and prevent future diseases
  - c) To monitor medications only
  - d) To discuss personal health concerns

1. **At what age is it generally advised to begin regular medical checkups for adults?**

- a) Childhood
  - b) 18-25 years
  - c) 30-40 years
  - d) 40+ years

**Section 2: Understanding Preventive Health Measures**

1. **How do regular checkups help in identifying chronic illnesses like diabetes or hypertension ?**
   - a) They help monitor the progression of existing conditions
   - b) They help to identify health risks before symptoms appear
   - c) They only manage current conditions
   - d) They are not necessary for chronic conditions
2. **Do you believe that mental health screenings should be included in standard routine checkups?**
   - a) Yes
   - b) No

**Section 3: Practices and Attitudes Toward Routine Checkups (these are opinion based questions so don’t have correct answers)**

1. **Do you personally schedule regular routine checkups even when you feel healthy?**
   - a) Yes
   - b) No
2. **Have you ever recommended routine checkups to family or friends as a preventive health measure?**
   - a) Yes
   - b) No
3. **When was the last time you had a routine medical checkup?**
   - Less than 6 months ago
   - 6-12 months ago
   - 1-2 years ago
   - More than 2 years ago
   - Never
4. **Do you feel confident in discussing the benefits of routine checkups with patients during your clinical rotations or practice?**
   - a) Yes
   - b) No
5. **Has your medical education influenced your attitude towards routine medical check-ups?**
   - Yes, positively
   - Yes, negatively
   - No influence
   - Not sure
6. **What factors influence your decision to schedule or not schedule a routine checkup? (Select all that apply)**
   - Health concerns or symptoms
   - Lack of time
   - Lack of health insurance or cost concerns
   - No perceived need (feel healthy)
   - Forgetfulness
   - Availability of healthcare facilities
   - Other (please specify): __________

**Section 4: Barriers Towards Routine Medical Checkups (these questions are also opinion based so no correct answers)**

12. **What do you think are the main barriers to routine medical checkups? (Select all that apply)**

- - Lack of time
  - Lack of awareness about the importance
  - Financial constraints or insurance issues
  - Fear of discovering health problems
  - Lack of healthcare providers in the area
  - Lack of trust in the healthcare system
  - Other (please specify): _______

13. **In your opinion, how can healthcare providers encourage the general population to undergo regular medical checkups? (Select all that apply)**

- - Offering reminders or health campaigns
  - Providing convenient and flexible appointment times
  - Offering affordable or free checkups
  - Providing more information about the benefits of routine checkups
  - Reducing waiting times at healthcare facilities
  - Other (please specify): ___________

***Thank you for participating in this questionnaire!***

**Section 2: Consent Form**

Informed Consent

| **Project Information** | |
| --- | --- |
| Project Title: Assessing Knowledge and Practices Towards Routine Medical Checkups Among medical students Of Karachi. |  |
| IRB Ref No: JSMU/IRB/2025/1025 | Sponsor: None |
| Principal Investigator: Laiba Akram | Organization: KMDC |
| Location: Block M North Nazimabad Town, Karachi, 74700 | Phone: :+92 3343449154 |
| Other Investigators:Muhammad Saad Khan,Dr Uzma Nasib, Zarmeen Azhar, Zunnerah Akram, Muhammad Mohsin Khan, Abeeha Naqvi | Organization: JSMU & KMDC |
| Location: V22W+F2H، Rafiqui H.J, Iqbal Shaheed Rd, Karachi Cantonment Karachi, Karachi City, Sindh 75510. | Phone: +92 347 8339460,+92 332 2376116 +92 334 3449154, +92 346 7096761, +92 322 2606087 |

.

# PURPOSE OF THIS RESEARCH STUDY

You are being asked to participate in a research study designed to assess the level of knowledge and practice regarding routine medical checkups among medical students in Karachi. The goal is to explore factors influencing the decision to undergo routine health evaluations and understand barriers to their practice. This data can help health professionals and policymakers in Karachi improve awareness and access to preventive healthcare.

# PROCEDURES

You will be asked to complete an online questionnaire that includes questions about your knowledge and practices regarding routine medical checkups. It will take approximately 10–15 minutes to complete. No physical procedures are involved.

# POSSIBLE RISKS OR DISCOMFORT

This study involves minimal risk. There is no expected physical or psychological harm.

# POSSIBLE BENEFITS

While there may be no direct benefit to you, the study will contribute valuable data to improve public health awareness and preventive care practices among medical students and possibly the general public.

# FINANCIAL CONSIDERATIONS

There is no financial compensation for participation in this study. Participation will not incur any additional cost to you.

# AVAILABLE TREATMENT ALTERNATIVES

Not applicable. This study does not involve treatment or intervention.

# AVAILABLE MEDICAL TREATMENT FOR ADVERSE EXPERIENCES

As the study does not involve any physical or medical procedures, medical treatment is not expected to be necessary. However, if you feel unwell during or after participation, you are encouraged to seek medical help.

# CONFIDENTIALITY

Your identity will remain confidential. Data will be anonymized using numeric codes. Results may be published, but no information that could identify you will be shared. Only the research team may have the access to raw data.

# RIGHTS TO PARTICIPATE, SAY NO, OR WITHDRAW

Participation is entirely voluntary. You may refuse to take part or withdraw from the study at any time without any penalty or loss of benefits.

# TERMINATION OF RESEARCH STUDY

You may choose to leave the study at any point. There are no consequences for withdrawal. The investigator may also terminate your participation if deemed necessary.

# AVAILABLE SOURCES OF INFORMATION

1. Any further questions you have about this study will be answered by the Principal Investigator:

Name: Laiba Akram

Phone Number: +92 334 3449154

1. Any questions you may have about your rights as a research subject will be answered by:

Name: Muhammad Saad Khan

Phone Number: +92 332 2376116

1. In case of a research-related emergency, call: Day Emergency Number: *+92 334 3449154*

Night Emergency Number: *+92 334 3449154*

# AUTHORIZATION

I have read and understood this consent form. I voluntarily agree to participate in this research study. I know that I can withdraw at any time. I understand that my legal rights are not affected by consenting to participate.

Name of participant (Printed or Typed): Date:

Signature of participant:

Date:

Signature of Principal Investigator:

Date:

Signature of person obtaining consent: Date:
